# Explaining local variation in referrals from health services to children’s social care in England 2013–16: a study using ‘children in need’ administrative data

**DOI:** 10.1093/pubmed/fdz050

**Published:** 2019-06-18

**Authors:** E H Emmott, L Mc Grath-Lone, K Harron, J Woodman

**Affiliations:** 1 UCL Anthropology, University College London, 14 Taviton Street, London, UK; 2 Rees Centre for Research in Fostering and Education, Department of Education, University of Oxford, 15 Norham Gardens, Oxford, UK; 3 UCL Great Ormond Street Institute of Child Health, University College London, 30 Guildford Street, London, UK; 4 Thomas Coram Research Unit, UCL Institute of Education, University College London, 27–28 Woburn Square, London, UK

## Abstract

**Background:**

Referral rates from Health service to Children’s Social Care (CSC) services vary across England. In 2019, the National Audit Office (re)iterated the urgent need to understand the drivers of such variation.

**Methods:**

Using administrative data (Children in Need Census, 2013–16), we calculated annual referral rates from Health to CSC services (Health referral rate) by Local Authority (LA) areas. We used multilevel linear regression to investigate the relationship between age-adjusted Health referral rates and local need (demand factors) and local practice/systems (supply factors). We present a tool to compare unadjusted and adjusted LA rates.

**Results:**

There was high LA variation in Health referral rates, particularly for infants (mean = 29.0/1000 children < 1 y; range = 6.5–101.8; sd = 12.4). LA variation persisted after age-adjustment. Child poverty (local need) and overall referral rate (local practice/systems) explained 60% of variation in age-adjusted Health referral rates. Overall referral rate was the strongest predictor. Adjusted referral rates were substantially different from unadjusted rates. After adjustment, 57.7% of LAs had higher/lower Health referral rates than expected.

**Conclusions:**

While higher levels of local need are associated with higher Health referrals, some areas have high Health referrals *irrespective of local need*. Our tool demonstrates the benefits of using adjusted rates to compare LAs.

## Introduction

Child abuse and neglect (maltreatment) is common, affecting approximately 1 in 10 children each year in the Global North, although estimates vary according to definitions and measures.^1^ Child maltreatment contributes to child mortality and morbidity, with wide-ranging and serious consequences that persist into adulthood.^1^ For these reasons, services and interventions to prevent child maltreatment and mitigate associated harms continue to be of high public health importance.^2–4^

In England, Children’s Social Care (CSC) is the local government agency tasked with coordinating, commissioning and delivering welfare interventions for children who require additional support to achieve a reasonable standard of health and development. A substantial minority of children are referred to CSC for ‘suspected child abuse or neglect’ (45%), ‘family dysfunction’ (18%), and ‘family in acute stress’ (10%), while a small proportion relate to complex health needs in the child or family (5%).^5^ This means that the vast majority of referrals to CSC are due to concerns about current or future harm to a child’s development and health. Referral to CSC is common: in 2017–18, 655,630 referrals were made (5.5 per 100 children),^6^ with one study estimating that one in five children (22.5%) are referred before their fifth birthday.^7^ This is broadly similar to figures from Australia and the USA.^7^ As others have summarized: the wide reach of CSC is only justifiable if it improves the lives of children and families; an outcome which is currently contested.^7,8^

English law and statutory government policy position Health services as a key agency in identifying and supporting children with additional welfare needs.^9–12^ Over the last decade, there has been increasing devolution of Health and Social Care to the 152 English Local Authorities (LAs; local government bodies), bringing increasing variation in how Health and CSC services work together.^13,14^ Variation brings opportunities to better meet local public health needs, but also increases the risks of inequalities in service quality, access, outcomes and joint-working practices.^15–17^ As the National Audit Office (NAO) recently argued, there is a need to understand geographical variation in CSC services across England to ensure that services are adequate and effective for children in need of help or protection, offer value for money, and are sustainable.^18^ The same report highlights the current evidence gap around geographical variation in CSC services, particularly in terms of quantifying and understanding what drives variation in CSC services.^18^ Our study contributes to this much needed evidence-base.

Variation of service provision between areas might be driven by differences in local need (‘demand factors’), and/or differences in the local service system and culture (‘supply factors’) and/or might be an artefact of the way that data is recorded locally.^19^ There is strong evidence of inequality in rates of child welfare interventions from 18 LAs in England^20^: poorer children have a higher chance of experiencing an intervention (structural inequality^21^) but children in similarly deprived circumstances faced unequal chances of intervention depending on where they lived (‘inverse care law’, or ‘post-code lottery’ of services^18,20^). Such inequality impedes the policy ambition for equity of care across local areas set out by The Department for Education in 2016.^22^

To date, studies on understanding variation in child welfare practices have focused primarily on CSC, rather than Health services. Recent government statistics indicate that referrals from Health to CSC services vary significantly across the 152 LAs in England, with Health contributing as little as 7% of all referrals in some areas and as high as 23% in others.^7^ However, we currently lack understanding of *why* such variation exists. The current government standard is to compare raw referral rates,^23^ which does not tell us whether variation can be explained by differences in local demand for services (i.e. differences in population need) or whether this is driven by differences in local practice and systems. Understanding variation is the first step in identifying principles of good practice which can be more widely shared.

## Objectives

We explore if and how local need (demand factors) and wider practice and system (supply factors) determine variations in joint-working between Health and CSC services, with specific focus on referral practice.

## Methods

### Data source

We use the Children in Need (CIN) census; a national, child-level administrative dataset of CSC case records in England held by the Department for Education (the central government body responsible for CSC services).^24^ CIN has been collected since October 2008, and information on referral source, including from Health, is available from first April 2013. Further detail on CIN is available elsewhere.^6^

Our CIN sample included 1372,352 children aged 0–17, with 1909,613 referrals between first April 2013 and 31st March 2016. We use the Office of National Statistics (ONS) mid-year child population estimates (0–17 years)^30^ to derive annual referral rates for each area (LA and Region). We estimate referrals rates from all sources, ‘Health referral rates’ (referrals specifically from health services), and referral rates from non-health sources (henceforth ‘overall referral rate’).

We take overall referral rate to capture the local referral practice beyond Health, reflecting a complex mix of local referral policy, practice and culture. Further, as an indicator of joint-working ‘quality’ between local agencies (including between Health and CSC), we use the most recently available Local Safeguarding Children’s Board (LSCB) inspection ratings. LSCBs are statutory agencies in each LA, responsible for cross-agency coordination to safeguard and promote the welfare of children.^25^ Their effectiveness is evaluated and rated by Ofsted.^26^ Overall referral rates and LSBC ratings are conceptualized as proxies of broader local practice in the area (i.e., ‘supply’ factors).^19^

As a proxy of LA population diversity, we include ONS 2016 mid-year estimates of percentage of people with White ethnicities, percentage British born, and percentage of British nationals in an area.^27^ As a measure of child poverty, we use snapshot data from HM Revenue & Customs on the percentage of children in low-income families in 2014 ( < 60% of the median income, or in receipt of state income support or income-based job seekers allowance).^28^ Local population composition and poverty are conceptualized as proxies of *local need* (or demand).^19,29^

Descriptive statistics of our LA-level data are available in SI1.1.

### Analysis methods

We describe national, regional and LA-level annual referral numbers and rates across England between 2013–16, including age-adjusted referral rates at regional and LA-level (standardized using ONS national population distribution of children aged 0–17 yrs).^30^ We also provide period-average rates over three years (2013–16).

We modelled the association between age-adjusted LA-level Health referral rates and LA-characteristics using linear regression with a multilevel structure to take account of repeat measures of referral rates within LAs across census years. Three LAs were excluded from our models: two due to small population size and lack of ONS population statistics, and one after initial exploration revealed anomalously high referral rates (see SI1.2). Our models included 447 Health referral rates from 149 LAs.

Model selection was informed by assessing Akaike Information Criteria (AIC).^31^ The best fitting model included percentage of children in poverty, overall referral rate, and LSCB rating. We compare how much variation in Health referral rates is explained by these predictors (percentage change in the intercept variance; %∆).^32^ All analyses were carried out in R v.3.4.3 using lme4 v.1.1–13^33^ (see SI1.5 for code and SI2 for data).

## Results

### Referrals to children’s service in England

On average, there were 54.5 referrals per 1000 children per year to CSC services (Table 1), and 7.6 referrals per 1000 children per year from Health services (range = 0.8–29.7; sd = 3.6; *N* = 152). Some children experienced multiple referrals per year, with an average of 1.15 referrals per child each year. On average, 47.2 *children* were referred per 1000 children each year (see SI1.1).

**Table 1:** Annual referral rates to children’s services between 2013 and 2016, by source and area.

**England**		**2013/14**			**2014/15**			**2015/16**			**2013–16**		
**Referral Source**		***N***	**% Referrals**	**Referral Rate (per 1 000 children)**	***N***	**% Referrals**	**Period Average Referral Rate (per 1 000 children)**	***N***	**% Referrals**	**Referral Rate (per 1 000 children)**	**Period Total *N***	**% Referrals**	**Period Average Referral Rate (per 1 000 children)**
All Referrals		661,608	100.0	57.1	640,896	100.0	54.9	607,109	100.0	51.5	1, 909,613	100	54.5
Health Services		88, 990	13.5	7.7	92, 923	14.5	8.0	84, 415	13.9	7.2	266,328	13.9	7.6
Police		154,425	23.3	13.3	167,055	26.1	14.3	168,573	27.8	14.3	490,053	25.7	14.0
Education		104,723	15.8	9.0	117,125	18.3	10.0	119,940	19.8	10.2	341,788	17.9	9.7
LA Services		88, 469	13.4	7.6	92, 533	14.4	7.9	89, 944	14.8	7.6	270,946	14.2	7.7
Individual		68, 100	10.3	5.9	61, 872	9.7	5.3	56, 035	9.2	4.8	186,007	9.7	5.3
Other		89, 803	13.6	7.7	81, 003	12.6	6.9	71, 964	11.9	6.1	242,770	12.7	6.9
Unknown		54, 201	8.2	4.7	25, 484	4.0	2.2	16, 238	2.7	1.4	95, 923	5	2.7
**Regions**		***All Referrals, 2013–16***						***Referrals from Health Services, 2013–16***					
		**Period Average Crude Annual Referral Rates (per 1 000 children)**			**Period Average Age-Adjusted Annual Referral Rates (per 1 000 children)**			**Period Average Crude Annual Health Referral Rates (per 1 000 children)**			**Period Average Age-Adjusted Annual Health Referral Rates (per 1 000 children)**		
**LA Regions**	**N LAs**	**Regional Referral Rates**	**LA Referral Rate Range**	**Relative Referral Risk (against EE)**	**Regional Referral Rates**	**LA Referral Rate Range**	**Relative Referral Risk (against EE)**	**Regional Referral Rates**	**LA Referral Rate Range**	**Relative Referral Risk (against EE)**	**Regional Referral Rates**	**LA Referral Rate Range**	**Relative Referral Risk (against EE)**
NE	12	61.2	38.2, 96.0	1.45	62	42.1, 92.2	1.45	10	6.0, 17.8	1.82	10.2	6.0, 17.5	1.84
NW	23	61.8	32.5, 114.6	1.46	62.4	31.1, 107.6	1.46	8.4	4.4, 18.1	1.53	8.5	4.0, 16.5	1.52
Y&H	15	64.9	34.1, 168.9	1.53	65.5	30.1, 163.1	1.53	9.5	4.5, 32.9	1.73	9.6	4.2, 29.7	1.73
EM	9	61.9	29.1, 107.1	1.46	62.6	29.8, 102.5	1.47	9	2.4, 16.7	1.64	9.1	2.6, 15.1	1.65
WM	14	59.8	33.0, 111.8	1.41	60.4	35.6, 96.3	1.41	7.6	3.4, 13.2	1.38	7.7	3.9, 12.0	1.38
EE	11	42.3	26.7, 55.8	1	42.7	26.0, 68.5	1	5.5	1.8, 8.3	1	5.5	2.3, 9.9	1
IL	14	52.1	18.7, 82.2	1.23	52.5	32.8, 85.1	1.23	8.4	0.6, 19.1	1.53	8.2	0.8, 19.2	1.47
OL	19	44.6	25.5, 65.7	1.05	44.9	25.0, 64.8	1.05	6.5	3.7, 12.2	1.18	6.5	3.6, 11.8	1.16
SE	19	50.9	30.5, 125.3	1.2	51.9	31.9, 98.5	1.22	6.7	2.6, 12.9	1.22	6.8	2.5, 11.3	1.23
SW	16	50	25.4, 101.3	1.18	50.7	24.7, 98.5	1.19	7.1	2.5, 17.3	1.29	7.2	2.9, 16.2	1.3

LA Regions are as follows: NE = North East; NW = North West; Y&H = Yorkshire & Humber; EM = East Midlands; WM = West Midlands; EE = East of England; IL = Inner London; OL = Outer London; SE = South East; SW = South West.Period average referral rates are mean annual referral rates between 2013 and 2016. Children are those aged 0–17 years.

Variation in referral rates persisted after age-adjustment. Within region, there was high variation in age-adjusted referral rates between individual LAs (Table 1; see SI2 for individual LA rates). For example, in Yorkshire & Humber, referral rates form individual LAs ranged from 4.2 to 29.7 per 1000 children (seven-fold difference).

**Fig. 1:**
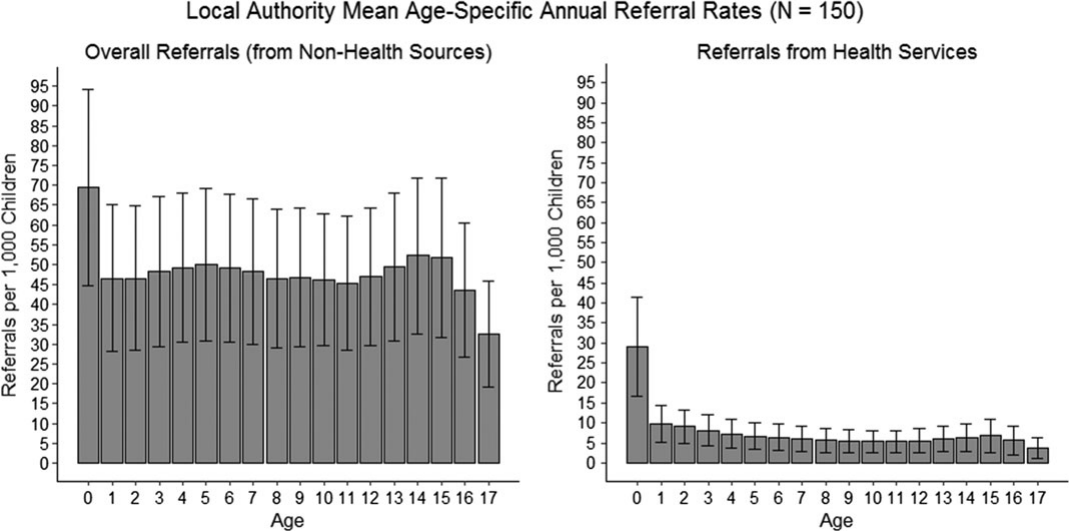
Annual age-specific referral rates, averaged across three years (2013/14 to 2015/16) for 150 Local Authorities. From our original sample of 152 Local Authorities, two were removed due to particularly small under-18 population sizes (under 2 500) and no recorded referrals for the majority of age groups. The error bars are standard deviations, representing spread of data (rather than standard errors).

For both non-Health and Health referrals, infants (age < 1 y) had the highest referral rates (Figure 1). Health referral rates were notably higher for infants compared to other age groups, and infant Health referral rates had the greatest variation between LAs (mean = 29.0 per 1000 children < 1 y; range = 6.5–101.8; sd = 12.4).

### Drivers of LA age-adjusted health referral rates

Child poverty, overall referral rate and LSCB rating explained 18.9%, 58.1% and 1.7% of LA-level variations in age-adjusted Health referral rates respectively (Table 2; see %∆ Intercept Variance). Higher area-level child poverty was associated with higher Health referral rates: a 6.7% increase in local proportion of child living in poverty (1sd change) was associated with 1.3 extra referrals from Health per 1000 children (Model 2).

**Table 2:** Descriptive statistics and model results for our final multilevel linear regression models with LA Health Referral Rate as the outcome.

Descriptive statistics			Model results						
				Model 1	Model 2	Model 3	Model 4	Model 5	Model 6
			N(LA) = 149 N(Observation) = 447	Null model	Adjusted for % child poverty (*z*)	Adjusted for overall referral rate (*z*)	Adjusted for LSCB Ofsted rating	Full model	Best fit model adjusted for child poverty + overall referral rate
	*N*			B (se)	B (se)	B (se)	B (se)	B (se)	B (se)
**Local authority**	149		**Intercept**	7.660 (0.287)	7.660 (0.269)	7.660 (0.207)	7.371 (0.447)	7.840 (0.312)	**7.660 (0.206)**
**Health referral rate**	Mean	SD							
2013/14	7.66	3.77	**Year**						
2014/15	7.68	3.61	2013/14 (ref)	–	–	–	–	–	–
2015/16	7.23	3.11	2014/15	0.022 (0.236)	0.022 (0.236)	0.022 (0.200)	0.022 (0.236)	0.022 (0.200)	**0.022 (0.200)**
			2015/16	−0.430 (0.236)	−0.430 (0.236)	−0.430 (0.200)	−0.430 (0.236)	−0.430 (0.200)	**−0.430 (0.200)**
**% Child poverty (in 2014)**	Mean	SD							
	21.03	6.70	**% Child Poverty (z)**	–	1.262 (0.233)	–	–	0.396 (0.181)	**0.400 (0.179)**
**Overall referral rate**	Mean	SD	**Overall Referral Rate (z)**	–	–	2.312 (0.136)	–	2.220 (0.143)	**2.217 (0.142)**
2013/14	49.95	18.71							
2014/15	47.78	19.66	**LSCB Ofsted Rating**						
2015/16	45.47	15.03	Good/Outstanding (ref)	–	–	–	–	–	–
			Requires Improvement	–	–	–	0.284 (0.568)	−0.485 (0.388)	–
**LSCB Ofsted rating**	*N*	%	Inadequate	–	–	–	1.317 (0.746)	0.354 (0.512)	–
Good/outstanding	52	34.9	Missing (not yet inspected)	–	–	--	−1.434 (1.322)	−0.609 (0.388)	–
Requires improvement	66	44.3							
Inadequate	25	16.8	Intercept Variance	8.150	6.613	3.412	8.013	3.298	**3.321**
Missing (not yet inspected)	6	4.0	Residual Variance	4.162	4.162	2.982	4.162	2.974	**2.974**
			%∆ Intercept Variance	–	18.9%	58.1%	1.7%	59.5%	**59.3%**
			AIC	2203.5	2179.5	1993.7	2200.2	1992.8	**1992.3**
			∆AIC	–	23.9	209.8	3.3	210.7	**211.2**

Note, all referral rates are age-adjusted. (*z*) indicates standardization by SD. LA = local authority; LSCB = Local Safeguarding Children Board; AIC = Akaike Information Criteria.

An increase in overall referral rate by 15–19 referrals (1sd change) was associated with 2.3 extra referrals from Health per 1000 children (Model 3). However, the independent effect of child poverty after controlling for overall referral rate was relatively small (Models 5 and 6): a 1sd increase in child poverty predicted 0.4 extra referrals per 1000 children. Overall, our best fit model included both child poverty and overall referral rate, explaining around 60% of LA variation in Health referral rates (Model 6).

In our data, poverty was moderately positively correlated with overall referral rate (*r* = 0.42), and a 1sd increase in child poverty was associated with 7.8 extra referrals in overall referral rate (see SI1.4.2). Our results suggest that the effect of child poverty on Health referral rate is mediated by overall referral rate. In other words, high child poverty is associated with a “wider system” high referrals in the area, which in turn predicts high referrals from Health. However, controlling for child poverty had minimal impact on the effect of overall referral rate on Health referrals. This suggests that local practice of higher overall referrals lead to high referrals from health, *over and beyond* child poverty.

**Fig. 2:**
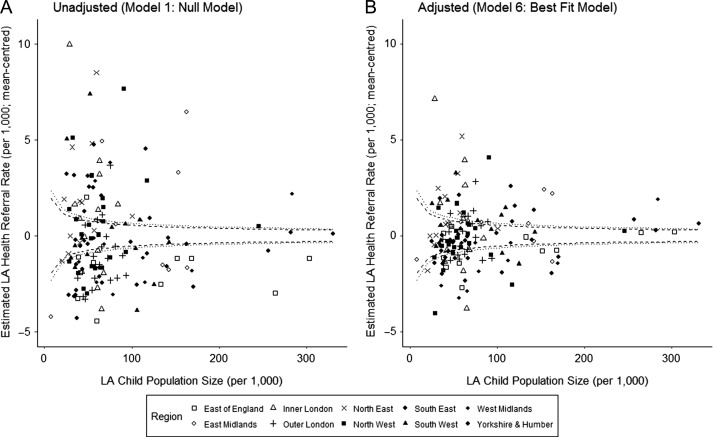
Funnel plots of (A) unadjusted and (B) adjusted LA Age-Adjusted Health Referral Rates in England, mean-centred with the England Mean. Unadjusted estimates are from the Null Model (Model 1 from Table 2) and adjusted estimates are from the Best Fit Model (Model 6 from Table 2), taking account of LA Child Poverty and Overall Referral Rates. Dotted lines indicate 95% and 98% control limits of the England Mean, calculated as outlined in Dover & Schopflocher (2011).^34^

80.5% of unadjusted LA Health referral rates (*N* = 120) fell outside the 95% control limits of the England mean (Figure 2, see SI3 for data). After adjusting for child poverty and overall referral rate, 57.7% of LA referral rates (*N* = 83) fell outside the 95% control limits of the England mean (i.e., had Health referral rates that were higher or lower than we would expect given their local poverty, overall referral rate and population size). A significant number of Health and CSC services are “behaving differently” regarding referrals, even after controlling for local need and the wider ‘non-health’ system.

## Discussion and Conclusions

### Main findings of this study

We found substantial variation in referral rates from Health to CSC between LAs, driven by particularly high variation in referrals for infants, but which persisted after age-adjustment. Child poverty was associated with higher Health referral rates. However, our strongest predictor was a measure which reflects the wider professional system in which Health is working (‘overall referral rate’) and which mediated the effect of child poverty on Health referral rate. This suggests that an area with higher referrals from all agencies tends to have higher referrals from health, over and above child poverty. It is also likely that the characteristics of local practice, system and culture is partly a response to local need: i.e. our ‘overall referral rate’ measure already captures area-level deprivation to a certain degree.

Following these findings, we hypothesized that the effect of overall referral rates on Health referral rates might differ according to the deprivation-level in the local area. For example, might Health services be ‘picking up’ extra unmet need in areas with high poverty but low overall referral rate (and thereby weakening the effect of poverty in our model)? We conducted a post-hoc analysis testing for an interaction effect between overall referral rate and poverty but we did not find support for this hypothesis (see SI1.4.1).

Overall, child poverty and overall referral rates explained 60% of the LA variation in age-adjusted Health referral rates. However, almost 57.7% of LAs had an adjusted Health referral rate that was significantly higher or lower than we would expect, controlling for population size. This suggests that the way Health services and CSC services are engaging around referrals is notably different between areas, even after taking local need and some wider system differences into account.

Adjusted Health referral rates for individual LAs were substantially different from unadjusted rates. Using our simple tool (SI4), LAs can see how their Health referral rates from 2015–16 compare to a) their statistical neighbours and b) other LAs in their region before and after adjusting for child poverty and overall referral rate. For example, this tool shows us that Blackpool had the highest crude Health referral rate among its statistical neighbours but after adjustment, they had the lowest. This changes the starting point for inspection bodies and service managers who wish to assess, understand and improve children’s services within a LA.

### What is already known on this topic

Health Services are key agencies in identifying and referring potentially vulnerable children with additional support needs to CSC.^25^ In England, raw referral rates from Health to CSC services vary notably between LAs.^7^ Previous studies suggest that variations in the rate of CSC interventions may be influenced by ‘demand factors’ (population need) and ‘supply factors’ (policy, practice and resource),^1,35–38^ with some evidence to suggest stronger influence of ‘supply’ factors.^17,18,20^ The relative impact of these drivers has not been quantified for referrals from Health to CSC services.

### What this study adds

Our results suggest that a significant number of Health and CSC services are “behaving differently” regarding referrals, even after controlling for local need and the wider local practice/systems. The fact that practice does not seem to be strongly driven by local need may reflect a so-called ‘postcode lottery’ of services around child welfare. Local variation in systems and practice can lead to innovation, improved services/outcomes and learning which might be shared. However, if child poverty rises at the rate that some predict^39^ and with shrinking resources,^40^ it may be increasingly important for Health and CSC services to better align their practice to local need.

Given the strong relationship between overall referral rate and Health referral rate, our results indicate that alignment of Health and CSC practice to local need might be achieved at least in part by focusing on the broader system (practice and culture) without targeting Health services specifically. As we also found that Health services and CSC services engage differently between LAs, over and above the wider system, targeted service change focusing on joint-working between Health and CSC services also has potential for success. Given that variation in Health referral rates is primarily due to variation in infant referrals, seeking to understand referral practice in maternity services and early health visiting is an obvious next step.

As recently as April 2019, the Department for Education has compared adjusted rates within groups of ‘statistical neighbours’ to identify LAs with higher than expected rates of CSC services (in this case, out-of-home care).^41^ However, our simple tool (SI4) illustrates that the standard practice of comparing unadjusted rates between LAs to understand practice is inappropriate.^23^ Unadjusted rates are useful mainly as a measure of relative workload. Using adjusted rates may better help the Department for Education, inspection bodies and internal service managers (such as Ofsted, LSCB and MASH-managers^42^ in England) to understand the workings of local Children’s Services. Adjusted rates could be used as a starting point to illicit a deeper understanding of differences in practice, systems and outcomes to facilitate self-learning, and highlight areas of good practice that might be shared.

### Limitations of this study

This study does not tell us what referral rates mean. Are higher than expected referral rates a signal of ‘good’ or ‘bad’ practice and systems? Should Health and CSC services be working to increase or decrease referrals? We do not know the answer. However, practice and context is too complex to dichotomise into ‘good’ and ‘bad’ based on a single statistic, such as higher- or lower-than-expected referral rates. It is likely that higher-than-expected rates in some LAs will be working well for children and families, while in others it will be a signal of poor joint-working/unwillingness for Health to take ownership of child safeguarding.

The lack of a ‘gold standard’ in practice is an enduring issue in child and family policy.^38^ Our study is a first step to better understand Health and CSC joint-working at a local level, but further studies are needed to understand the contexts in which higher- and lower-than-expected referral rates occur, and in which contexts the system is functioning well. To address this, we are currently undertaking a qualitative study with three LA sites, to better understand the local mechanisms and practice around unexpectedly high/low Health referral rates. This is an increasingly important question given increasing demand and shrinking resources,^40^ with some evidence that this is leading to higher thresholds for intervention (the “severity” at which CSC services assess children and/or offer services) and a focus on short-term intervention, with potential for higher service failure (no resolution of child and family problems, evidenced by re-referrals within a short time frame^29^).

There are also some issues with our data. First, we have limited proxy measures of demand and supply factors. Our measure of ‘demand’ factors is crude, as child poverty alone is unlikely to accurately reflect the aggregate ‘need’ within the LA. This could weaken the association between ‘demand factors’ and health referral rate in our analyses. Equally, our proxy of local practice (‘supply factors’) is blunt. While the LSCB rating is a measure of joint-working quality provided by Ofsted, information on actual practice at LA level is not systematically available. We assume overall referral rate reflects a complex construct of local referral culture and practice, but our study is unable to reveal what aspects of referral culture and practice is influencing Health referral rates. For example, it is possible that the variation in referral rates is driven by referral *recording* rather than referral *behaviour* (i.e. variation could be an ‘artefact’ of the data).

Secondly, we used a LA-level outcome (referral rate) because CIN data does not contain any information on children not referred. To estimate risk of referral from Health to CSC for different groups of children, CIN data would need to be linked to other population data. Such a linkage would allow more nuanced analyses, including to investigate if children with health conditions are more likely to be referred to CSC by Health than other children. This could be extended to include analyses of parents/carers with health conditions who are also likely to be coming into higher than average contact with Health services and have extra demands on them that may impact parenting capacity. With more granular data, we could also explore how household, neighbourhood and LA-level deprivation influence Health referral rates. CIN currently does not include any information on household level deprivation or need (e.g., parental characteristics), which is limiting.

Thirdly, the lack of metadata around how LAs are recording referral sources and some issues with data quality means it is possible that some referrals from Health sources have been incorrectly recorded as ‘other’ or ‘missing’.^6^ While we cannot know the extent of any coding error, if the issue is prevalent we may be underestimating the effect of overall referral rate on Health referral rate.

Further studies are needed to better understand both recording practices and differences in systems and professional practice: we are beginning this process with a linked qualitative study underway which seeks to understand the variation we report in this paper.

## Supplementary Material

fdz050_SI1_AdditionalInfo_V2

fdz050_SI2_FinalModelData

fdz050_SI3_FunnelPlotData_V2

fdz050_SI4_StatisticalNeighboursTool
