# Supplementary material for: Explaining local variation in referrals from health services to children’s social care in England 2013–16: a study using ‘children in need’ administrative data
Source: J Public Health (Oxf). 2019 Jun 18;43(1):180–8. doi: 10.1093/pubmed/fdz050 (PMC8042370; doi:10.1093/pubmed/fdz050)
Supplement: fdz050_SI1_AdditionalInfo_V2 [file fdz050_si1_additionalinfo_v2.docx]

# Supplementary Information 1

## SI1.1 Additional descriptive statistics

The following supplementary information (Table SI1) provides additional descriptive statistics on Local Authority referrals and children referred, and variables used in our initial analyses which were subsequently excluded from the final models. Note, the population size and characteristic variables were explored by a thousandth and z-scored.

*Table SI1: Additional Descriptive Statistics*

| **Time-Varying Variables** | | | | **Time-Static Variables** | | |
| --- | --- | --- | --- | --- | --- | --- |
|  | **2013/14** | **2014/15** | **2015/16** |  |  |  |
| **N Health Referrals** |  |  |  | **% Child Poverty (2014)** |  |  |
| LA Mean | 586.1 | 606.1 | 553 | LA Mean | 21.03 |  |
| LA Upper Quartile | 745 | 721 | 709 | LA Upper Quartile | 25.85 |  |
| LA Median | 431 | 423 | 444 | LA Median | 21.18 |  |
| LA Lower Quartile | 267 | 281 | 265 | LA Lower Quartile | 15.57 |  |
| LA SD | 514.1 | 568.2 | 428.1 | LA SD | 6.70 |  |
| **N Children Referred from Health** |  |  |  | **% White Ethnicity (Total population estimate; 2016)** |  |  |
| LA Mean | 562 | 579.2 | 535 | LA Mean | 75.60 |  |
| LA Upper Quartile | 706 | 680 | 692 | LA Upper Quartile | 91.49 |  |
| LA Median | 418 | 411 | 431 | LA Median | 83.94 |  |
| LA Lower Quartile | 255 | 271 | 262 | LA Lower Quartile | 65.99 |  |
| LA SD | 487.4 | 527.6 | 407.9 | LA SD | 21.33 |  |
| **N Non-Health Referrals** |  |  |  | **% British Nationality (Total population estimate; 2016)** |  |  |
| LA Mean | 3791 | 3771 | 3824 | LA Mean | 88.93 |  |
| LA Upper Quartile | 4582 | 4542 | 1879 | LA Upper Quartile | 95.05 |  |
| LA Median | 2693 | 2724 | 2767 | LA Median | 92.34 |  |
| LA Lower Quartile | 1842 | 1867 | 4537 | LA Lower Quartile | 84.65 |  |
| LA SD | 3058.8 | 3033.2 | 3143.1 | LA SD | 8.44 |  |
| **N Children Referred from Non-Health** |  |  |  | **% UK Born (Total population estimate; 2016)** |  |  |
| LA Mean | 2936 | 3016 | 2994 | LA Mean | 82.94 |  |
| LA Upper Quartile | 3314 | 3557 | 3371 | LA Upper Quartile | 92.35 |  |
| LA Median | 2341 | 2417 | 2290 | LA Median | 88.04 |  |
| LA Lower Quartile | 1546 | 1592 | 1630 | LA Lower Quartile | 77.23 |  |
| LA SD | 2244.3 | 2278.6 | 2156.4 | LA SD | 13.36 |  |
| **Health Referral Rate** |  |  |  |  |  |  |
| LA Mean | 7.76 | 7.75 | 7.76 |  |  |  |
| LA Upper Quartile | 9.84 | 9.26 | 8.71 |  |  |  |
| LA Median | 7.11 | 7.10 | 6.98 |  |  |  |
| LA Lower Quartile | 5.02 | 5.13 | 5.24 |  |  |  |
| LA SD | 3.80 | 3.66 | 3.13 |  |  |  |
| **Non-Health Referral Rate** |  |  |  |  |  |  |
| LA Mean | 49.89 | 49.52 | 49.08 |  |  |  |
| LA Upper Quartile | 60.32 | 60.62 | 60.48 |  |  |  |
| LA Median | 45.78 | 45.14 | 44.54 |  |  |  |
| LA Lower Quartile | 36.35 | 35.89 | 35.63 |  |  |  |
| LA SD | 16.69 | 18.63 | 18.54 |  |  |  |
| **Time-Varying Variables, Continued** | | | | **Time-Static Variables, Continued** | | |
| **Age-adjusted Health Referral Rate** |  |  |  |  | **N** | **%** |
| LA Mean | 7.66 | 7.68 | 7.23 | **Ofsted Ratings:** |  |  |
| LA Upper Quartile | 9.80 | 9.30 | 8.71 | *Children in Need* |  |  |
| LA Median | 6.96 | 7.00 | 6.78 | Outstanding | 0 | 0 |
| LA Lower Quartile | 4.83 | 5.16 | 5.17 | Good | 36 | 24.16 |
| LA SD | 3.77 | 3.61 | 3.11 | Requires Improvement | 77 | 51.68 |
| **Age-adjusted Non-Health Referral Rate** |  |  |  | Inadequate | 30 | 20.13 |
| LA Mean | 49.96 | 47.78 | 45.47 | Missing | 6 | 4.03 |
| LA Upper Quartile | 60.55 | 54.40 | 52.90 | *Management and Governance* |  |  |
| LA Median | 45.36 | 43.20 | 43.16 | Outstanding | 10 | 6.71 |
| LA Lower Quartile | 36.68 | 34.85 | 35.13 | Good | 43 | 28.86 |
| LA SD | 18.71 | 19.66 | 15.03 | Requires Improvement | 60 | 40.27 |
| **N Population Under 18** |  |  |  | Inadequate | 30 | 20.13 |
| LA Mean | 77,324 | 77,898 | 78,612 | Missing | 6 | 4.03 |
| LA Upper Quartile | 89,902 | 90,805 | 91,941 | *Local Safeguarding Children Board* |  |  |
| LA Median | 59,703 | 59,614 | 60,806 | Outstanding | 5 | 3.56 |
| LA Lower Quartile | 42,465 | 42,616 | 42,981 | Good | 47 | 31.54 |
| LA SD | 57,671 | 57,968 | 58,381 | Requires Improvement | 66 | 44.30 |
| **N Population Under 5** |  |  |  | Inadequate | 25 | 16.78 |
| LA Mean | 22,885 | 22,909 | 22,870 | Missing | 6 | 4.03 |
| LA Upper Quartile | 28,043 | 28,309 | 27,887 |  |  |  |
| LA Median | 17,937 | 17,984 | 18,062 |  |  |  |
| LA Lower Quartile | 12,571 | 12,636 | 12,485 |  |  |  |
| LA SD | 16,354 | 16,351 | 16,323 |  |  |  |
| **N Total Population** |  |  |  |  |  |  |
| LA Mean | 362,248 | 365,379 | 368,587 |  |  |  |
| LA Upper Quartile | 418,269 | 420,585 | 422,727 |  |  |  |
| LA Median | 269,076 | 274,022 | 277,962 |  |  |  |
| LA Lower Quartile | 198,294 | 200,996 | 202,220 |  |  |  |
| LA SD | 271,356 | 273,368 | 275,614 |  |  |  |

## SI1.2 Exploratory Analyses

Our initial exploration of LA Health Referral Rates suggested there were some outliers with slight negative skew (some LAs having very high LA Health Referral Rate; see Figure SI1).

| A) | B) | C) |
| --- | --- | --- |
| 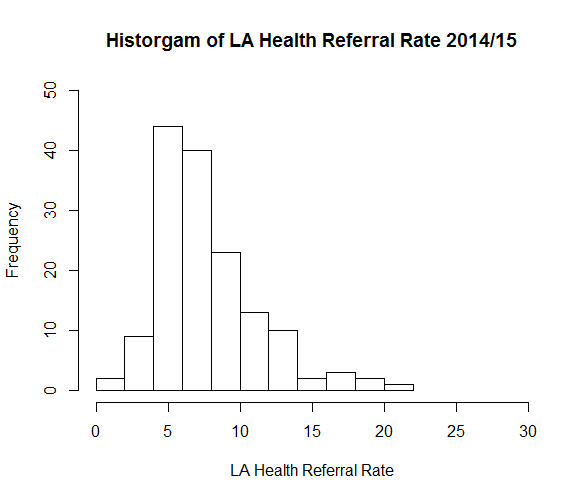 | 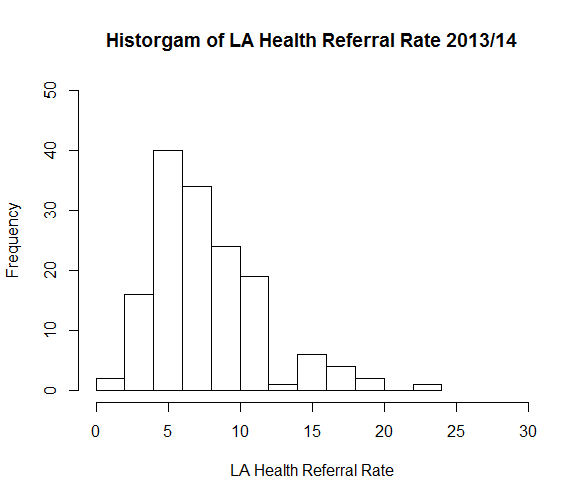 | 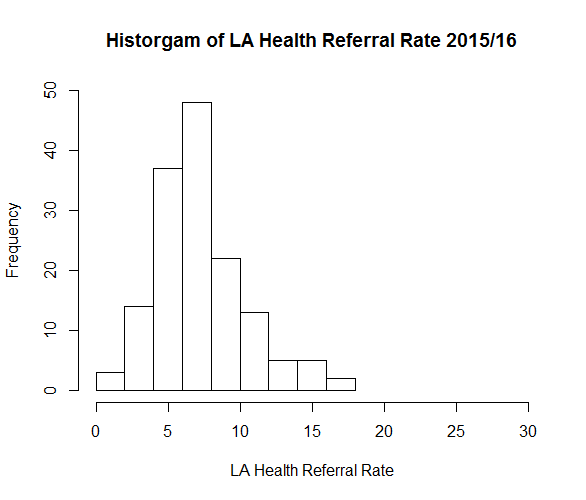 |

Figure SI1 Histograms of LA Health Referral Rates per census year, A) 2013/14, B) 2014/15, and C) 2015/16.

We then ran null models and examined Q-Q plots or random intercepts and conditional residuals (Figure SI2) which suggested that a one LA was a notable outlier. We therefore removed this outlier.

| A) | B) |
| --- | --- |
| 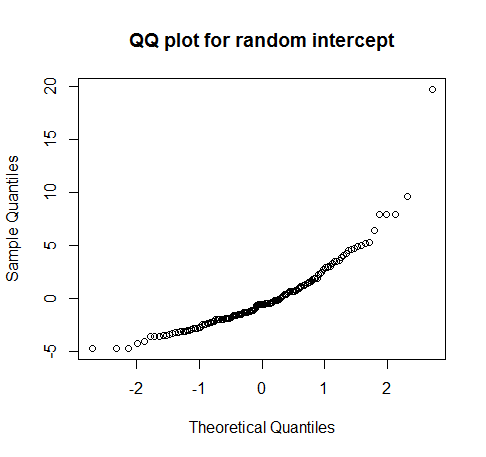 | 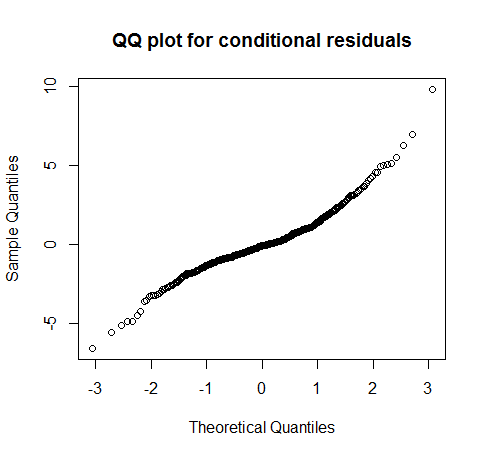 |

Figure SI2 QQ plots of our initial null model with LA Health Referral Rate as dependent variable and Census Year as predictor with random intercept. N(LA)=150.

## SI1.3 Model Selection

As part of our model selection process, we explored possible determinants of LA Health Referral Rates by adding each candidate predictor to the null model. If the addition of the predictor variable increased the AIC value (indicating worse model fit), it was assumed not to be an important determinant of LA Health Referral Rates and excluded from further analyses. Table SI2 outlines AIC scores of all predictor variables included in this model selection process.

*Table SI2: AIC scores of models, for addition of each predictor variable to the NULL model. N(LA)=149.*

| **Predictor Variable** | **AIC** | **Change in AIC** | **Decision** |
| --- | --- | --- | --- |
| NULL (reference model) | 2203 | NA | NA |
| + Ofsted LSCB Judgement | 2200 | -3 | Included |
| + Overall Referral Rate (from non-health sources), Z-scored | 1994 | -209 | Included |
| + Proportion of people with while ethnicities in LA | 2212 | +9 | Excluded |
| + Proportion of people with British nationalities in LA | 2210 | +7 | Excluded |
| + Proportion of people born in the UK in LA | 2212 | +9 | Excluded |
| + Under-18 population size in LA | 2214 | +11 | Excluded |
| + Total population size in LA | 2217 | +14 | Excluded |
| + Child Poverty in LA, z-scored | 2180 | -23 | Included |

## SI1.4 Post-Hoc Analyses

We conducted post-hoc analyses to help us infer our results around Child Poverty.

### SI1.4.1 Interaction between Child Poverty and Overall Referral Rate

Following on from our best fit model presented in our main paper, we specifically tested whether the effect of child poverty interacted with the effect of overall referral rate. We hypothesised that, in areas with high poverty but low overall referral rate, health services could be ‘picking up’ extra referrals to children’s social care. We did not find evidence to support this hypothesis, and the interaction effect between child poverty and overall referral rate did not improve model fit (Best fit model AIC= 1993; Interaction model AIC=1996). The results are outlined below (Table SI3).

*Table SI3: Results from post-hoc model, testing interaction effects between child poverty and overall referral rate.*

| N(LA)=149  N(Observation)=447 | **Best Fit Model** | **Interaction Model** |
| --- | --- | --- |
|  | B (se) | B (se) |
|  |  |  |
| **Intercept (mean health referral rate)** | 7.660 (0.206) | 7.629 (0.215) |
|  |  |  |
| **Year** |  |  |
| 2013/14 (ref) | -- | -- |
| 2014/15 | 0.022 (0.200) | 0.028 (0.200) |
| 2015/16 | -0.430 (0.200) | -0.428 (0.199) |
|  |  |  |
| **% Child Poverty (z)** | 0.400 (0.179) | 0.406 (0.181) |
|  |  |  |
| **Overall Referral Rate (z)** | 2.217 (0.142) | 2.201 (0.145) |
|  |  |  |
| **Child Poverty * Overall Referral Rate** | **--** | **0.074 (0.140)** |
| Intercept Variance | 3.321 | 3.382 |
| Residual Variance | 2.974 | 2.962 |
| **AIC** | **1992.3** | **1996.2** |
| **∆AIC** | **--** | **+3.9** |

### SI1.4.2 Exploring the relationship between Overall Referral Rate and Child Poverty

To better understand the relationship between child poverty and overall referral rate, we conducted an additional analysis investigating the association between LA child poverty (z-scored) and overall referral rate. As before, we carried out a multi-level model with overall referral rate as the dependent variable, with census year as level 1 and LA as level 2. In our model, a 1sd increase in LA child poverty predicted just under 8 extra referrals from non-health sources in a Local Authority area (Table SI4).

*Table SI4: Key results from interaction model between child poverty and overall referral rate.*

| N(LA)=149  N(Observation)=447 | **B (se)** |
| --- | --- |
| **Intercept (mean overall referral rate)** | 49.886 (1.389) |
|  |  |
| **Year** |  |
| 2013/14 (ref) | -- |
| 2014/15 | -0.363 (0.050) |
| 2015/16 | -0.803 (0.050) |
|  |  |
| **% Child Poverty (z)** | 7.836 (1.393) |
|  |  |
| Intercept Variance | 287.103 |
| Residual Variance | 0.181 |

## SI1.5 R code for multilevel models

For transparency, we provide the R code to replicate our main multilevel models presented in the manuscript. Data is available from our Supplementary Information (SI2_FinalModelData.xlsx). To run the code, you need R (free download from <https://www.r-project.org/>) and we recommend using R Studio (free to download from <https://www.rstudio.com/>). **Once you have opened R, copy and paste the code below into your R Script window. Text after ‘#’ are comments or instructions.** The data provided also allows to replicate the post-hoc analyses presented in this document (SI1.4). We do not provide data for the LAs excluded from analyses, partly as the number of referrals a low increasing risk of individual identification (as part of standard disclosure control; [see the national pupil database agreement for the supply of data, p.10](https://dera.ioe.ac.uk/19740/1/Agreement_for_the_supply_of_NPD_data_-_Sample.pdf)).

*#1.Set your working directory folder & make sure SI2_FinalModelData.xlsx is saved there*

*#2.Load required packages. You must first install these packages if you do not have them installed in R.*

*library(readxl)*

*library(lme4)*

*#3. Load data for multilevel regression models*

*mydata<-read_excel("SI2_FinalModelData.xlsx", sheet=2)*

*#4. Deriving variables*

*#4.1 To help interpretation of model outputs, we convert population size variables into per 1,000 and mean-centre for each census year*

*#Under-18 Population Size*

*#divide by 1000*

*mydata$Under18_k_centre<-mydata$N_Under18/1000*

*#center by time*

*for(i in 0:2) {*

*mydata$Under18_k_centre[mydata$Time==i]<-scale(mydata$Under18_k_centre[mydata$Time==i], center=T,scale=F)*

*}*

*#Total Population Size*

*#divide by 1000*

*mydata$TotalPop_k_centre<-mydata$N_TotalPop/1000*

*#center by time*

*for(i in 0:2) {*

*mydata$TotalPop_k_centre[mydata$Time==i]<-scale(mydata$TotalPop_k_centre[mydata$Time==i], center=T,scale=F)*

*}*

*#4.2 We also centre population composition.*

*#PWhite*

*mydata$PWhite_centre<-scale(mydata$PWhite, center=T,scale=F)*

*#PUKborn*

*mydata$PUKborn_centre<-scale(mydata$PUKborn, center=T, scale=F)*

*#PBritish*

*mydata$PBritish_centre<-scale(mydata$PBritish, center=T, scale=F)*

*#4.3 We also z-score Child Poverty and Non-Health Referrals (overall referral rate in manuscript). We do this for each census period.*

*#Child Poverty*

*mydata$ChildPoverty_z<-mydata$ChildPoverty*

*for(i in 0:2) {*

*mydata$ChildPoverty_z[mydata$Time==i]<-scale(mydata$ChildPoverty_z[mydata$Time==i], center=T,scale=T)*

*}*

*#Non-Health Referral Rate*

*mydata$NonHealthRefRate_Z<-mydata$NonHealthReferralRate_Adjusted*

*for(i in 0:2) {*

*mydata$NonHealthRefRate_Z[mydata$Time==i]<-scale(mydata$NonHealthRefRate_Z[mydata$Time==i], center=T,scale=T)*

*}*

*#4.4 We change time & LSCB into categorical variables. LSCB Good and Outstanding are recategorised into 1 category due to the small number of Outstanding LSCBs.*

*mydata$Time<-as.factor(mydata$Time)*

*mydata$Ofsted_LSCB[mydata$Ofsted_LSCB=="Good" | mydata$Ofsted_LSCB=="Outstanding"]<-"Good/Outstanding"*

*mydata$Ofsted_LSCB<-as.factor(mydata$Ofsted_LSCB)*

*#5. Model Selection*

*#Null*

*m1<-lmer(HealthReferralRate_Adjusted ~ Time + (1 | LA_ID), data=mydata)*

*summary(m1) #This gives you the model results*

*AIC(m1) #This shows you the AIC score of the null model*

*#+ Under 18 pop size*

*m2<-lmer(HealthReferralRate_Adjusted ~ Time + Under18_k_centre + (1 | LA_ID), data=mydata)*

*#+ Total pop size*

*m3<-lmer(HealthReferralRate_Adjusted ~ Time + TotalPop_k_centre + (1 | LA_ID), data=mydata)*

*#+ Proportion White*

*m4<-lmer(HealthReferralRate_Adjusted ~ Time + PWhite_centre + (1 | LA_ID), data=mydata)*

*#+ Proportion UK-born*

*m5<-lmer(HealthReferralRate_Adjusted ~ Time + PUKborn_centre + (1 | LA_ID), data=mydata)*

*#+ Proportion British*

*m6<-lmer(HealthReferralRate_Adjusted ~ Time + PBritish_centre + (1 | LA_ID), data=mydata)*

*#+ Child Poverty*

*m7<-lmer(HealthReferralRate_Adjusted ~ Time + ChildPoverty_z + (1 | LA_ID), data=mydata)*

*#+ Non-Health referrak rate*

*m8<-lmer(HealthReferralRate_Adjusted ~ Time + NonHealthRefRate_Z + (1 | LA_ID), data=mydata)*

*#+ LSCB*

*m9<-lmer(HealthReferralRate_Adjusted ~ Time + Ofsted_LSCB + (1 | LA_ID), data=mydata)*

*#Compare AIC(i)-AIC(NULL). We take AIC change <=-3 as evidence that the additional parameter is likely to be a better model (see Burnam & Anderson 2000, p.70 )*

*for(i in 2:9) {*

*if((AIC(get(paste("m",i, sep="")))-AIC(m1))<=-3) print(paste("Include variable from m",i, sep=""))*

*}*

*#6. Final models, as outlined in Table 2 of the manuscript.*

*#Model 1 - Null Model*

*summary(m1)*

*AIC(m1)*

*#Model 2 - Adjusted for % Child Poverty (Z)*

*summary(m7)*

*AIC(m7)*

*#Model 3 - Adjusted for Overall Referral Rate (z)*

*summary(m8)*

*AIC(m9)*

*#Model 4 - Adjusted for LSCB Ofsted Rating*

*summary(m9)*

*AIC(m8)*

*#Model 5 - Full Model*

*m10<-lmer(HealthReferralRate_Adjusted ~ Time + ChildPoverty_z + NonHealthRefRate_Z + Ofsted_LSCB + (1 | LA_ID), data=mydata)*

*summary(m10)*

*AIC(m10)*

*#Model 6 - Best Fit Model*

*m11<-lmer(HealthReferralRate_Adjusted ~ Time + ChildPoverty_z + NonHealthRefRate_Z + (1 | LA_ID), data=mydata)*

*summary(m11)*

*AIC(m11)*
